# Genetic variants and phenotype analysis in a five-generation Chinese pedigree with *PCDH19* female-limited epilepsy

**DOI:** 10.3389/fneur.2023.1107904

**Published:** 2023-03-09

**Authors:** Wenjuan Zhou, Yuzhen Ouyang, Yuqiao Ji, Qiong Xi, Lingling Zhao

**Affiliations:** ^1^Third Xiangya Hospital, Central South University, Changsha, Hunan, China; ^2^Xiangya School of Medicine, Central South University, Changsha, Hunan, China

**Keywords:** *PCDH19-FE*, phenotype heterogeneity, pedigree, *PCDH19* variants, epilepsy

## Abstract

**Objective:**

Albeit the gene of *PCDH19*-FE was ascertained, the correlation of gene mutation, *PCDH19* protein structure, and phenotype heterogeneity remained obscure. This study aimed to report a five-generation pedigree of seven female patients of *PCDH19*-FE and tried to explore whether two variants were correlated with *PCDH19* protein structure and function alteration, and *PCDH19*-FE phenotype.

**Methods:**

We analyzed the clinical data and genetic variants of a *PCDH19*-FE pedigree, to explore the phenotype heterogeneity of *PCDH19*-FE and underlying mechanisms. In addition to the clinical information of family members, next-generation sequencing was adopted to detect the variant sites of probands with validation by sanger sequencing. And the sanger sequencing was conducted in other patients in this pedigree. The biological conservation analysis and population polymorphism analysis of variants were also performed subsequently. The structure alteration of mutated *PCDH19* protein was predicted by AlphaFold2.

**Results:**

Based on a five-generation pedigree of *PCDH19*-FE, missense variants of c.695A>G and c.2760T>A in the *PCDH19* gene were found in the heterozygous proband (V:1), which resulted in the change of amino acid 232 from Asn to Ser (p.Asn232Ser) and amino acid 920 from Asp to Glu (p.Asp920Glu) influencing *PCDH19* function. The other six females in the pedigree (II:6, II:8, IV:3, IV:4, IV:5, IV:11) exhibited different clinical phenotypes but shared the same variant. Two males with the same variant have no clinical manifestations (III:3, III:10). The biological conservation analysis and population polymorphism analysis demonstrated the highly conservative characteristics of these two variants. AlphaFold2 predicted that the variant, p.Asp920Glu, led to the disappearance of the hydrogen bond between Asp at position 920 and His at position 919. Furthermore, the hydrogen bond between Asp920 and His919 also disappeared when the Asn amino acid mutated to Ser at position 232.

**Conclusion:**

A strong genotype-phenotype heterogeneity was observed among female patients with the same genotype in our *PCDH19*-FE pedigree. And two missense variants, c.695A > G and c.2760T>A in the *PCDH19* gene, have been identified in our pedigree. The c.2760T>A variant was a novel variant site probably related to the *PCDH19*-FE.

## Introduction

As one of the six genes most involved in genetic epilepsy, protocadherin 19 (*PCDH19*), located at Xq22.1, was highly expressed in multiple organs especially the limbic areas of the brain, which was involved in cell adhesion, neuronal migration, synaptic connections formation (1). With a six-exon structure, *PCDH19* encoded protocadherin-19, a transmembrane cell adhesion molecule belonging to the cadherin family participated in the neuronal connections (2). The first exon encoded the extracellular, transmembrane, and a small portion of the intracellular domain. The rest of the C-terminal intracellular domain was encoded by exons 2–6, and cytoplasmic domains (CM1 and CM2) in the C-terminal region were encoded by exons 5 and 6. *PCDH19* female limited epilepsy (*PCDH19*-FE) was an infantile-onset epilepsy syndrome with strong phenotype heterogeneity, and hallmark features of *PCDH19*-FE included focal seizure, intellectual disability, anti-seizure therapy resistance, etc. (3). *PCDH19*-FE was also named an epilepsy-intellectual disability in females, which was first reported in 1971 (4). However, its causal gene wasn't identified until 2008 based on seven families (5). In addition to heterozygous females, *PCDH19*-FE also affected male mosaicism but the male hemizygotes were exempt (6). To date, ~150 variants in *PCDH19* have been discovered, which contained non-sense, missense, and frameshift variants (7). Among these variants, missense variants were mostly reported, of which c.1019A > G (p.Asn340Ser) heterozygous variant was one of the common types (8). Besides, while a high variant frequency was found in the extracellular domain (EC) of *PCDH19*, the variant in the intracellular domain impacting signal transduction was also reported (5, 7). In addition to the genetic variant diversity, the phenotypic spectrum of the disease varied widely including mild epilepsy to epileptic encephalopathy. Seizure types also ranged from focal seizures to generalized tonic-clonic, tonic, absence, and myoclonic seizures (9). With clustered and fever-sensitive attack characteristics, neurodevelopmental disorders such as autism spectrum disorder (ASD), and attention-deficit hyperactivity disorder (ADHD) may also occur (10). Albeit the heterogeneity of variant and phenotype has been summarized, a convincible explanation for this genotype-phenotype heterogeneity still lacked. In this article, we reported a pedigree of *PCDH19*-FE including five generations, seven patients in the pedigree. The same variant and strong heterogeneity of clinical symptoms were found in this pedigree. A novel variant was identified and potential explanations of strong phenotype heterogeneity compared with symptoms in previous cases and genotype-phenotype heterogeneity were also discussed in detail.

## Materials and methods

### Participants

**Figure 1:**
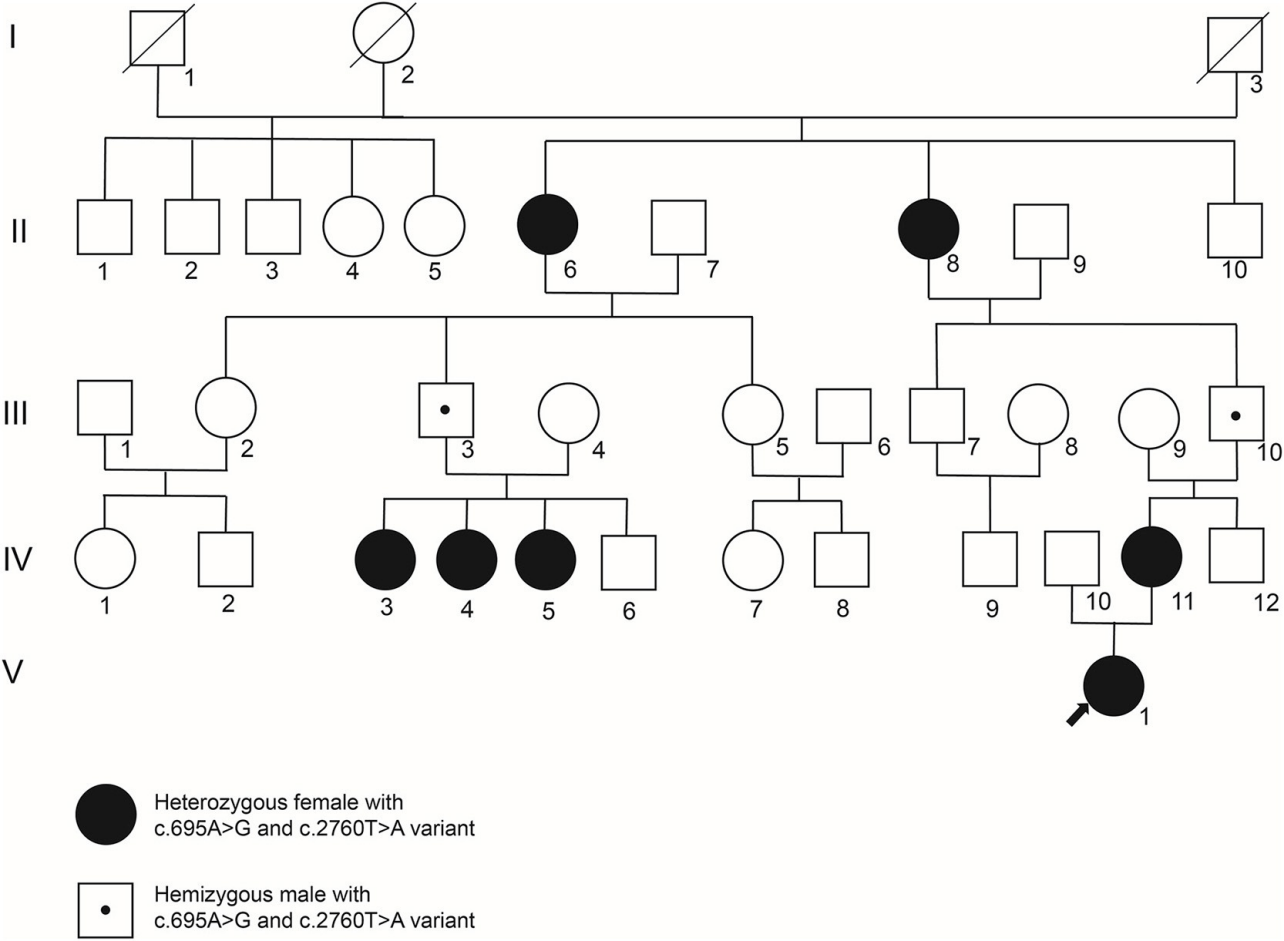
The genogram of a five-generation *PCDH19*-FE pedigree. While filled circles represent female patients, empty squares or circles represent unaffected males or females, respectively. The empty square or circle with an oblique line denotes a deceased male or female. And an empty square with a small point symbolizes an unaffected male who is a carrier of *PCDH19* c.695A>G and c.2760T>A variants. Blood samples of II:6, II:8, III:3, III:4, III:9, III:10, IV:3, IV:4, IV:5, IV:6, IV:11, IV:12, and V:1 were collected for genetic variant detection.

A five-generation Chinese pedigree with *PCDH19*-FE was analyzed in this study (Figure 1). All patients and other unaffected family members were evaluated by two independent pediatric neurologists from the Third Xiangya Hospital. A typical mode of inheritance was suggested in this pedigree as only female heterozygotes were affected whereas males hemizygotes were unaffected. Clinical data and venous blood samples were obtained from seven affected individuals (II:6, II:8, IV:3, IV:4, IV:5, IV:11, V:1) and six unaffected family members (III:3, III:4, III:9, III:10, IV:6, IV:12). This study was approved by the Institutional Review Board of the Third Xiangya Hospital of Central South University, and all participants signed consents to participate in the study.

### Proband-only whole exome sequencing and sanger sequencing

Two milliliter venous blood from patients was collected in Ethylenediaminetetraacetic acid (EDTA)–anticoagulated tube. The genomic DNA was extracted using FlexiGene DNA kits (Qiagen, product number 51206), and the ultra-micro spectrophotometer Nanodrop 2000 (Thermo Fisher Scientific) was utilized to assay the content of the DNA. To identify the disease-causing gene, whole exome sequencing (WES) in the proband (V1) was performed based on Agilent's SureSelect Human All Exon V5 Kit on the Illumina HiSeq 2000 platform (Novogene Bioinformatics Technology Co., Ltd). Qubit 2.0 (Life, product number 1719158) was applied to examine the content of the DNA sample in the captured library. Then, the library was then examined by IDT Bioanalyzer 2100 and loaded on the NEXTSEQ500 (Illumina) for sequencing. Then, the sequencing quality was assessed using a series of bioinformatics software including BWA, Samtools, Picard, and Genome Analysis Toolkit (GATK). Factors including the total count of reads, the percentage of reads that match the human genome sequence, the percentage of reads that locate inside the target sequence of the target gene, average sequencing depth, and coverage uniformity were assessed. Next, annotation was added to the genetic variants using PolyPhen-2.2.2 software, Annovar software, human gene mutation database (HGMD) database, dbSNP database, and 1000 Genome database. Lastly, selected variants were verified by Sanger sequencing.

Sanger sequencing was also performed in other family members (II:6, II:8, III:3, III:4, III:9, III:10, IV:3, IV:4, IV:5, IV:6, IV:11, IV:12) to identify potential disease-causing variant. Polymerase chain reaction (PCR) was adopted to amplify targeted exonic regions and intronic regions nearby. Sequences of the primers used for amplifying the causative variant of the PCDH19 (NM_001184880) were as follows: 5-TGGAGTCGATTGCTGCAACTT-3 (forward) and 5 -CGACATCATCCTGGCTCAGA-3 (reverse) for chrX:99596989 (hg19), 5-CTGACGGTGACCTTGCAGTG-3 (forward) and 5 -CCGAACTCGTGGTGGAAAAG-3 (reverse) for chrX:99662901 (hg19).

### *In silico* analysis and interpretation of *PCDH19* variants

The transcription version of PCDH19 used for genetic analysis was the NM_00184880.2. The Basic Local Alignment Search Tool (http://blast.st-va.ncbi.nlm.nih.gov/Blast.cgi) was used to conduct conservation analysis. And the frequency of the variant in the population was identified based on gnomAD, 1000 genomes, and the dbSNP database.

Further pathogenicity analysis was performed to predict the impact of variants, p.Asn232Ser and p.Asp920Glu. The score from SIFT (https://sift.bii.a-star.edu.sg/index.html), Mutation taster (https://www.mutationtaster.org/), and PolyPhen-2 (http://genetics.bwh.harvard.edu/pph2/) were adopted for the protein-damage prediction (11–13).

In combination with variant analysis and previous publications, p.Asn232Ser and p.Asp920Glu variants were interpreted based on the American College of Genetics and Genomics (ACMG) 2015 guideline, respectively (14, 15).

### 3D protein structure prediction by AlphaFold2

The state-of-the-art protein 3D structure prediction tool, AlphaFold2, was employed (https://colab.research.google.com/github/sokrypton/ColabFold/blob/main/beta/AlphaFold2_advanced.ipynb) to modal the spatial structure of wild-type and mutated *PCDH19* protein (16). Based on amino acid sequence before and after mutation, the subsequent protein structure prediction was conducted. The acquired prediction results from AlphaFold 2 were then visualized on UCSF Chimera with 3D protein structure diagram generation (http://www.cgl.ucsf.edu/chimera/) (17).

## Results

### Clinical findings

As shown in Figure 1, a five-generation pedigree was collected and analyzed. The symptoms of the proband (V:1) began at 19 months, and the ictal form was characterized by febrile generalized tonic-clonic seizure (GTCS). Clusters of seizures occurred with or without fever in the presence of respiratory tract infection. In addition to febrile and afebrile GTCS, the seizure form can also present as the tonic seizure with normal magnetic resonance imaging (MRI) manifestation. The electroencephalogram (EEG) result showed normal background rhythm, and no significant abnormal discharges during interictal periods. The tonic-clonic seizure of generalized origin or the tonic seizure of focal origin were recorded during ictal periods. The proband was treated with sodium valproate combined with levetiracetam but the seizure could not be completely controlled with mild developmental delay. When the proband was 6 years old, the follow-up was conducted, and an EEG examination was repeated exhibiting diffuse slow waves in the EEG background with a high discharge index during the inter-ictal period. The Gesell developmental scale indicated mild intellectual disability. In combination with clinical manifestations including slowing speech rate, epileptic encephalopathy was considered.

Febrile and afebrile GTCS have been observed among six other female patients. A wide spectrum of phenotypes including different psychiatric comorbidities, or different degrees of developmental delay was also identified in these patients. IV:3, IV:4, and IV:5 had tried sodium valproate and carbamazepine but the seizure was not controlled well. And they did not take anti-seizure drugs regularly due to economic reasons. As for IV:11, the seizure only appeared during fever, but she also didn't take any anti-seizure drugs regularly. Unfortunately, the medications and some other information about II:6 and II:8 were incomplete because of their ages. More detailed clinical information was summarized in Table 1. While the first seizure age varied from 8 to 48 months, the intellectual disability level is from mild to severe damage. As for language competence, while II:6, II:8, IV:3, IV:11, and V:1 kept the normal language ability, the language delay has been observed on IV:4 and IV:5. Autism was the most common psychiatric comorbidity in our pedigree, which has been diagnosed on IV:4, IV:5, and IV:11. EEG examinations were only conducted at the first visit of these patients. With normal inter-ictal EEG, epileptic seizures were recorded during the ictal period. In summary, a strong phenotype heterogeneity has been identified in our pedigree.

**Table 1:** Clinical manifestations of patients in this pedigree.

**Number**	**Age at study (years)**	**Seizure onset**		**Fever sensitivity**	**Status epilepticus**	**Cluster seizures**	**MRI findings**	**Intellectual disability**	**Language delay**	**Psychiatric comorbidities**
		**Age (months)**	**Type**							
II:6	68	Unknown	Unknown	Yes	No (after 17)	No (after 17)	/	No	No	Schizophrenia
II:8	66	Unknown	Unknown	Unknown	No	No	/	No	No	No
IV:3	14	30	GTCS	Yes	No	Yes	Normal	Moderate	No	No
IV:4	12	19	GTCS	Yes	No	Yes	Normal	Severe	Yes	Autism
IV:5	10	8	GTCS	Yes	No	Yes	Normal	Severe	Yes	Autism
IV:11	22	48	GTCS	Yes	No	Yes	Normal	Mild	No	Autism
V:1	1.8	19	GTCS	Yes	No	Yes	Normal	Mild	No	No

GTCS, generalized tonic-clonic seizures; MRI, magnetic resonance imaging.

### Sequencing result and analysis of variants

**Figure 2:**
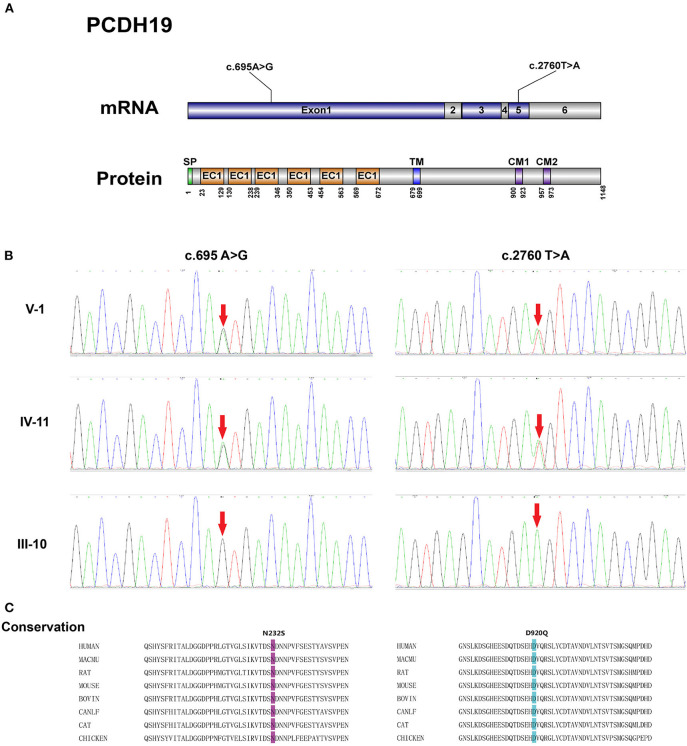
**(A)** Schematic representation of the structure of *PCDH19* mRNA and protein with two gene mutation sites identified in our pedigree. SP, signal peptide; EC, extracellular cadherin domain; TM, transmembrane domain; CM 1 and CM 2, cytoplasmic domains 1 and 2. **(B)** Electropherograms of Sanger sequencing of the *PCDH19* mutations: c.695A>G and c.2760T>A. **(C)** Alignment of the p.N232S and p.D920Q mutations among *PCDH19* orthologs in different species.

Blood samples of II:6, II:8, III:3, III:4, III:9, III:10, IV:3, IV:4, IV:5, IV:6, IV:11, IV:12, and V:1 were collected. Next-generation sequencing was performed on the proband (V:1) with variants filtered by the methods described above. As shown in Figure 2, two missense variants in *PCDH19* were identified, and the c.2760T>A variant was a novel variant site without reports before. The c.695A>G and c.2760T>A variants were located at exon 1 and exon 5, respectively. Sanger sequencing confirmed the c.695A>G and c.2760T>A heterozygous variants on the *PCDH19* gene in the proband V:1, which were inherited from her mother (IV:11) and grandfather (III:10). These two variants led to p.Asp920Glu and p.Asn232Ser alteration, which are located in the CM1, and extracellular domain2 (EC2) of *PCDH19* protein, respectively. And the c.695A>G and c.2760T>A variants were also both detected by sanger sequencing in II:6, II:8, III:3, III:10, IV:3, IV:4, IV:5, IV:11. However, no variant was detected in III:4, III:9, IV:6, and IV:12.

The conservation analysis of Asn232 and Asp920 amino acid residue was conducted by aligning protein sequences from different species included in the uniport database. Results from these tools demonstrate the highly conservative characteristics of these two variants. And the pathogenicity of the p.Asp920Glu and p.Asn232Ser were predicted by multiple *in silico* analysis techniques including SIFT, Polyphen2, and Mutation Taster. As shown in Table 2, the pathogenicity of these two variants was predicted as damaging or probably damaging. As for the variant frequency in the population, the c.2760T>A variant was not recorded in the gnomAD database (v 3.1.1). The c.695A>G variant was once detected in a European female sample and recorded in the gnomAD database (v 3.1.1) with the allele frequency of 8.84e-6 in the total population.

**Table 2:** Sequencing result and interpretation of the PCDH19 variant.

**Variants**	**Reported/novel**	**Chromosome position**	**Exon**	**SIFT**	**Mutation taster**	**PolyPhen-2**	**Interpretation (ACMG)**
c.695A>G (p.Asn232Ser)	Reported	chrX:99662901 (hg19)	Exon 1	D (0)	D (1)	PD (0.999)	Pathogenic (PS2 + PM1 + PM2 + PM5 + PP3 + PP5)
c.2760T>A (p.Asp920Glu)	Novel	chrX:99596989 (hg19)	Exon 5	D (0.001)	D (1)	PD (0.999)	Uncertain significance (PM1 + PM2 + PP3)

D, damaging or deleterious; PD, probably damaging.

### Interpretation of variants based on ACMG guideline

Based on the above analysis and previous publications, these two variants were interpreted based on the ACMG guideline (Table 2). The same variant of c.695A>G leading to the same amino acid change has been interpreted as pathogenic/likely pathogenic on the ClinVar database (Accession: VCV000206321). And the c.695A>G have been previously identified as *de novo* in previous pedigrees (3, 18–20). Besides, missense changes at the same codon (p.Asn232Ile and p.Asn232Lys) have been reported as pathogenic/likely pathogenic on the ClinVar database (Accession: VCV000159561, VCV000206322, VCV001068161). Therefore, the c.695A>G variant was interpreted as pathogenic (PS2 + PM1 + PM2 + PM5 + PP3 + PP5), and the novel c.2760T>A variant was classified as uncertain significance (PM1 + PM2 + PP3).

### Structure prediction of *PCDH19* protein before and after the mutation

**Figure 3:**
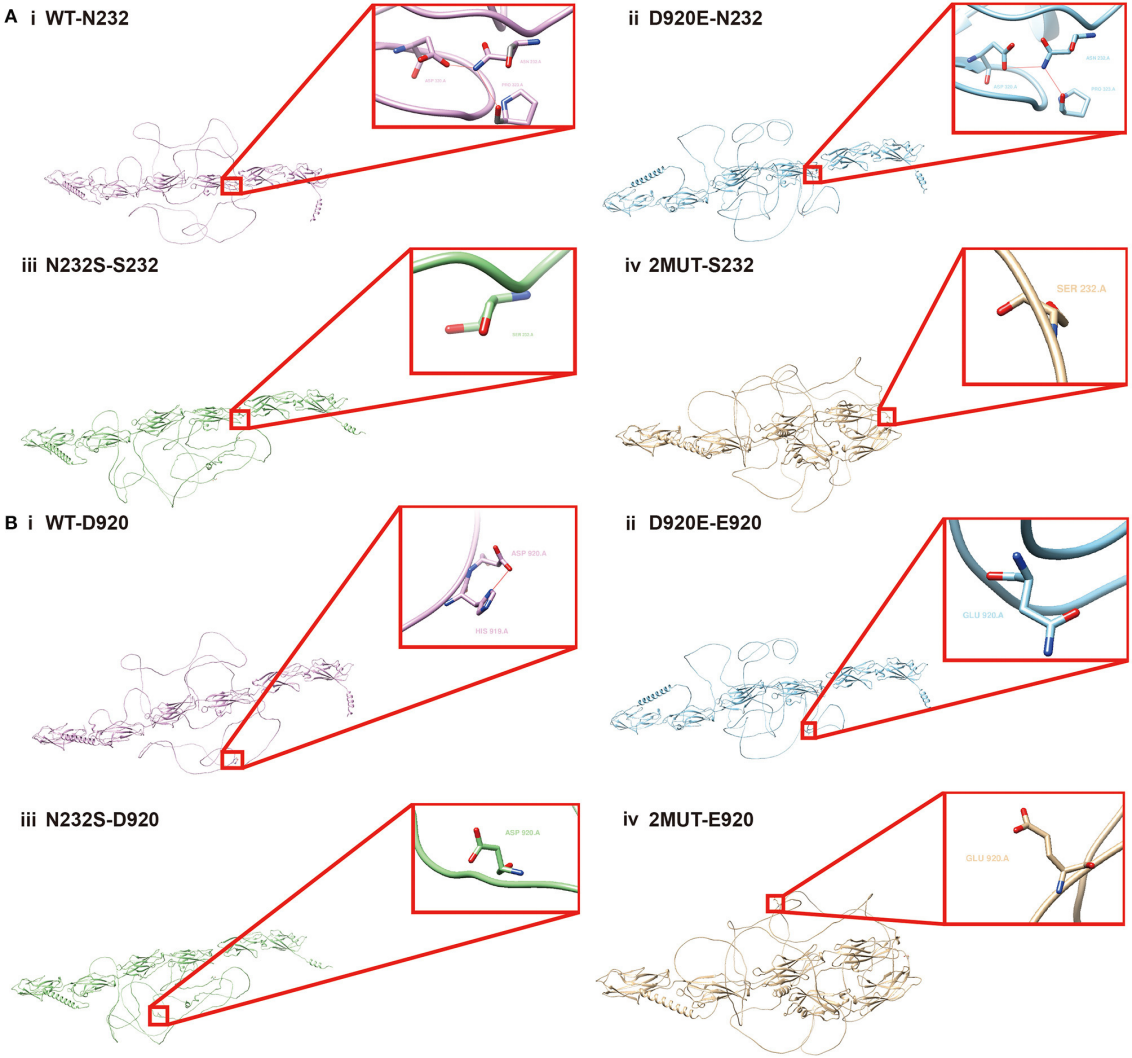
Structural alteration analysis of *PCDH19* protein by Alphafold2. N232S in the figure symbolizes Asn at the 232 amino acid position of *PCDH19* protein mutates to Ser, and the D920E represents the Asp at the 920 position mutates to Glu. **(A)** The *PCDH19* protein structure prediction and partially amplified conformation of the 232 amino acid position on *PCDH19* protein under the condition of (i) wild type, (ii) D920E, (iii) N232S, and (iv) the concurrent D920E and N232S mutation were exhibited. **(B)** The PCDH19 protein structure and partially amplified conformation of 920 amino acid position on the *PCDH19* protein under the condition of (i) wild type, (ii) D920E, (iii) N232S, and (iv) D920E and N232S mutation coexistence were also predicted.

Two variants in the *PCDH19* protein, p.Asn232Ser and p.Asp920Glu, probably interfered with the protein structure and its related function. It is also of concern whether there was mutual interaction between concurrent two variants, which may bring different effects on protein structure and function than the presence of one variant alone. Therefore, AlphaFold 2, one of the cutting-edge protein structure prediction tools, and UCSF Chimera were employed for *PCDH19* structure prediction. As shown in Figure 3A, in the wild-type protein, hydrogen bonds formed among Asn at position 232, Asp at position 320, and Pro at position 323, which disappeared when Asn at position 232 mutated to Ser. Figure 3B also exhibited that the hydrogen bond formed between Asp at position 920 and His at position 919 disappeared when the amino acid at position 920 was mutated to Glu. Furthermore, when the Asn amino acid mutated to Ser at position 232, the hydrogen bond between Asp920 and His919 also disappeared indicating the coexistence of p.Asp920Glu and p.Asn232Ser probably led to the altered intermolecular bond compared with that with a single variant alone. Therefore, the interplay by simultaneous variants in *PCDH19* was also correlated with altered intermolecular bonds of *PCDH19* protein. Whether the phenotype heterogeneity was correlated with interactions among different variant sites required further research.

## Discussion

In this research, a five-generation *PCDH19*-FE pedigree with strong genotype-phenotype heterogeneity was collected and analyzed. In addition to the comprehensive analysis of the pedigree with epilepsy and developmental delay from the clinical perspective, the sequencing of epilepsy-related genes was performed to investigate *PCDH19* genotype and phenotype heterogeneity. Two *PCDH19* variants in two loci, p.Asn232Ser and p.Asp920Glu, were identified. Besides, AlphaFold2 was adopted for mutated *PCDH19* protein structure prediction, which revealed these two variants, and the mutual interaction between these two variants was correlated with *PCDH19* structure alterations.

*PCDH19* was a member of the delta-cadherin (δ-cadherin) superfamily genes, and the δ-cadherin superfamily included a family of differentially expressed neuro-adhesion molecules involved in a range of neurodevelopmental disorders (21). *PCDH19* was predominantly expressed in the nervous system, especially in the developing limbic system, and was essential for calcium-dependent cell-cell interactions and adhesion (22). The increased expression level of *PCDH19* was also found in other areas such as the ventromedial telencephalon, thalamus, periventricular zone of the anterior hypothalamus, and numerous sensory and motor nuclei. Multiple variants in *PCDH19* have been discovered, which contained missense, non-sense, frameshift variants, etc. Variants can cause changes in protein structure and function in a variety of ways, including interference of homophilic adhesion, impairment of interaction with other calcium adhesion proteins, and a decrease in protein folding function and stability (23). Recent experiments on animal models illustrated that the *PCDH19* variant impaired the mossy fiber synapse development in a mouse model, and led to neuronal hyperexcitability in both mosaic and non-mosaic *PCDH19* mutation zebrafish models (24, 25). The pathophysiology of *PCDH19*-related epilepsy has also been reviewed recently with four main theories: gamma-aminobutyric acid type A receptor [GABAA(R)] dysregulation, cellular interference, blood-brain barrier dysfunction, and the aldo-keto reductase 1C family 1-3 (AKR1C1-3) gene product shortage (26). While GABAA(R) mediated the inhibitory postsynaptic potential, the AKR1C1-3 product deficiency led to the decreased allopregnanolone, which was correlated with decreased tonic and phasic inhibitory currents of GABAA(R) (27). As for the blood-brain barrier (BBB) dysfunction, increased substances such as antibodies of *N*-methyl-D-aspartate (NMDA)-type glutamate receptor (abs-NR) can pass the BBB, which may underlie disease pathogenesis (8). Furthermore, contrary to the loss function hypothesis of *PCDH19*, the cellular interference theory indicated that clinical manifestations may be related to abnormal communication between normal and mutant cells (28). This hypothesis was also supported by the phenomenon that mutated females and mosaic males were mostly affected while hemizygous males were commonly unaffected. And the correlation of X inactivation and *PCDH19*-FE phenotype may also be partly explained by this hypothesis.

From the clinical perspective, the *PCDH19* variant caused a spectrum of clinical symptoms of *PCDH19*-FE (23). While some shared clinical features such as seizures characterized by fever sensitivity, and early-onset seizures in infancy or early childhood, the phenotype heterogeneity was strong (3, 29, 30). Multiple seizure types have been observed such as GTCS, myoclonic, and absence seizures (31). The concomitant psychiatric symptoms were also diverse including hyperactive, autistic, and obsessive-compulsive features (7). In addition, epileptic encephalopathy was also reported in previous *PCDH19*-FE pedigree (32, 33).

In our pedigree, a strong genotype-phenotype heterogeneity was exhibited in the result part and Table 1. The clinical characteristic that symptoms of *PCDH19*-FE were observed in heterozygous females but not in hemizygous males was consistent with previous reports. In addition, the clinical manifestations of five generations in our pedigree seemed to become more severe in the newer generation. And patients with early onset tended to suffer from more severe symptoms. The clinical symptoms of V:1 with epileptic encephalopathy manifestations were more severe than those of IV:11. The intellectual disability was observed on IV:3, IV:4, and IV:5 but was not identified on II:6. Although there have been some reports of genotype-phenotype heterogeneity, mechanisms were still vague. Age was one of the influencing factors of *PCDH19*-FE as seizures commonly became less severe with growth (29). And onset age and seizure frequency were prognostic factors of intellectual disability (7). Besides, while most patients appeared cognitive delay after the epilepsy symptoms, 15% of patients exhibited cognitive delay before the epilepsy onset. The intellectual impairment was probably related to genetic factors, environmental factors, etc. In addition, the X chromosome inactivation was also a potential reason for phenotype heterogeneity. Previous publications revealed the association of X-inactivation and *PCDH19*-related epileptic encephalopathy with skewed X-chromosome inactivation as a potential protective factor (32). Epigenetic modifications participated in the skewed X-inactivation, which preferentially inhibited the expression of wild-type or mutant proteins returning the heterozygous to the homozygous state. Based on the cellular interference hypothesis, the comparable wild-type cells and mutant cells interacted with each other probably impacting the neurodevelopment or synaptogenesis (34, 35). And the skewed X-inactivation may reverse this pathogenic status reducing seizures and disorders.

Besides, *PCDH19* variants in two loci were identified in our research, and the p.Asn232Ser has been reported in previous cases of the clinical diagnosis of PCDH19-related epilepsy or Dravet syndrome with phenotype heterogeneity (3, 18–20, 33, 36). In addition to the common symptoms including cluster and febrile seizures, generalized seizures such as absence seizure and GTCS and focal seizure have been reported in multiple patients with the p.Asn232Ser variant (3, 18, 36). Intellectual disability, language, and motor delay were also reported (18, 20). Another female patient with the p.Asn232Ser variant typically began with hypomotor-apneic semiology mostly followed by tonic-clonic activity with posterior discharge on ictal EEG in infancy. Later, after an aura of lower extremity or abdominal pain, and myoclonia, tonic-clonic clusters and hypermotor seizures were observed (19). Intriguingly, the p.Asn232Ser variant has been detected in a European (non-Finnish) female sample but was not detected in the east Asian population based on the record on the gnomAD database (v3.1.1). The allele frequency of this variant was 8.84e-6 in the total population indicating that it can be detected in the asymptomatic person but was not a common variant in the population. Numerous cases of asymptomatic carrier mothers were also reported (37–39). As for the mechanism of the existence of asymptomatic carriers, the penetrance rate and X-inactivation are potential influencing factors. The penetrance of PCDH19-FE was 80% approximately but was still uncertain because of the indefinite definition of unaffected individuals and the estimation based only on reported cases with tested mothers (7). Previous parts have also discussed that the X-inactivation may involve in the phenotype heterogeneity, and the skewed X-inactivation was probably related to unaffected heterozygous female carriers. However, further researches were still necessary to obtain more accurate explanations.

The possible cause of the clinical phenotype heterogeneity between our cases and previous reports was probably associated with the novel p.Asp920Glu variant found in our pedigree. This p.Asp920Glu variant was highly conserved in biological species conservation and was not a polymorphic change in the population with a low frequency of occurrence. The pathogenicity of the p.Asp920Glu variant was testified by multiple *in silico* analyses in Table 2 including Polyphen2 and Mutation Taster. And the influence of p.Asp920Glu on *PCDH19* protein structure has also been predicted by AlphaFold2. The hydrogen bond formed between Asp at position 920 and His at position 919 disappeared when the amino acid at position 920 was mutated to Glu. As a membrane protein, *PCDH19* consisted of six extracellular cadherin repeats, a transmembrane domain (TM), and a C-terminal intracellular tail with two motifs, CM1 and CM2 (40). More than one hundred variants and a variety of variant types led to structural alterations in different domains of the *PCDH19* protein based on previous publications (7). And novel variants were also increasingly reported, which were mostly located at exon 1 and missense variations (7). Various variant types were recorded including missense/non-sense variants, small deletions, splicing variants, etc. However, the association of different missense variant sites and phenotype heterogeneity has not been summarized before but several publications have reported the correlation among gene variant sites, protein structure, and phenotype. For instance, a milder intellectual disability and later onset seizures were found in the patients with truncating variants from EC domain 5 to the cytoplasmic domain compared with truncating variants from EC domain 1 to EC domain 4 (22). As c.2760T>A was a novel variant located at the exon 5 of the *PCDH19* gene influencing the structure of the CM1 domain of *PCDH19* protein, the phenotype differences between our pedigree and previous cases were probably related to this variant requiring further experiments to verify this hypothesis. From the function perspective, *PCDH19* functions were also diverse such as its adhesive or signal transduction properties with potential influence on structural layering on hippocampus layering (41, 42). A complete *PCDH19* ectodomain model was put forward recently, which can assist in the evaluation of epilepsy-related variants and protein interaction sites (43). Diverse variants probably affected different functions of the *PCDH19* protein to varying degrees leading to heterogeneity. Besides, the mutual interactions between different mutated amino acids may also exert an influence on the structure and function of *PCDH19*. Our research revealed the amino acid alteration at position 230 led to the disappearance of the hydrogen bond between His919 and Asp920. This indicated that the interplay among simultaneous variants at different sites in *PCDH19* also probably impacted the intermolecular interaction of *PCDH19* protein.

In addition to our findings, there were a few limitations that existed in our research. First, the mechanism of genotype-phenotype heterogeneity, which has been observed in our pedigree and previous reports, requires further research to explore such as the X chromosome inactivation mode. Second, the functional verification on the animal model should be subsequently conducted to ascertain whether the p.Asp920Glu variant influences the *PCDH19* protein function and *PCDH19*-FE phenotype.

## Conclusion

Based on this *PCDH19*-FE pedigree with five generations, two variants, p.Asn232Ser and p.Asp920Glu in the *PCDH19*, were identified, of which p.Asp920Glu was a novel variant site without being reported before. And a strong genotype-phenotype heterogeneity has been observed among female patients with the same genotype in our pedigree. As for the potential mechanism of this genotype-phenotype heterogeneity, multiple factors including age, and X-chromosome inactivation mode were possible related factors but still needed further investigation. Other factors related to phenotype heterogeneity such as variant sites and the mutual interaction between concurrent variants in *PCDH19* were also discussed based on the genotype and clinical manifestations of patients in our pedigree and previous cases.

## Article summary

Two variants in *PCDH19* were identified in seven female patients with different clinical phenotypes in a five-generation pedigree with *PCDH19*-FE. And the c.2760T>A (p.Asp920Glu) variant is a novel variant site probably related to *PCDH19*-FE.

## Data availability statement

The datasets presented in this study can be found in online repositories. Our sequencing data have been deposited to the NCBI Sequence Read Archive (SRA) database of the national center for biotechnology information, and the accession number of it is PRJNA859639.

## Ethics statement

The studies involving human participants were reviewed and approved by Third Xiangya Hospital. Written informed consent to participate in this study was provided by the participants' legal guardian/next of kin. Written informed consent was obtained from the individual(s), and minor(s)' legal guardian/next of kin, for the publication of any potentially identifiable images or data included in this article.

## Author contributions

WZ and YO were involved in the conception and design of the study and the drafting of the manuscript. YJ participated in the drafting of the manuscript. WZ, QX, and LZ reviewed the manuscript for its intellectual content and revised the entire work. All authors approved the final manuscript as submitted and agree to be accountable for all aspects of the work.
